# New Quaternary Ammonium Derivatives Based on Citrus Pectin

**DOI:** 10.3390/polym15234492

**Published:** 2023-11-22

**Authors:** Magdalena-Cristina Stanciu, Marieta Nichifor, Gabriela-Liliana Ailiesei, Irina Popescu, Gabriela-Elena Hitruc, Luminita Ghimici, Cristina G. Tuchilus

**Affiliations:** 1 “Petru Poni” Institute of Macromolecular Chemistry, 41A Gr. Ghica-Voda Alley, 700487 Iasi, Romania; nichifor@icmpp.ro (M.N.); gdarvaru@icmpp.ro (G.-L.A.); ipopescu@icmpp.ro (I.P.); gabihit@icmpp.ro (G.-E.H.); lghimici@icmpp.ro (L.G.); 2 Faculty of Medicine, “Grigore T. Popa” University of Medicine and Pharmacy, 16 University Street, 700115 Iasi, Romania; ctuchilus@yahoo.com

**Keywords:** citrus pectin, quaternary ammonium pendant groups, self-assembly, antimicrobial activity

## Abstract

New citrus pectin derivatives carrying pendant *N*,*N*-dimethyl-*N*-alkyl-*N*-(2-hydroxy propyl) ammonium chloride groups were achieved via polysaccharide derivatization with a mixture of *N*,*N*-dimethyl-*N*-alkyl amine (alkyl = ethyl, butyl, benzyl, octyl, dodecyl) and epichlorohydrin in aqueous solution. The structural characteristics of the polymers were examined via elemental analysis, conductometric titration, Fourier Transform Infrared spectroscopy (FTIR) and 1D (^1^H and ^13^C) nuclear magnetic resonance (NMR). Capillary viscosity measurements allowed for the study of viscometric behavior as well as the determination of viscosity–average molar mass for pristine polysaccharide and intrinsic viscosity ([η]) values for pectin and its derivatives. Dynamic light scattering measurements (DLS) showed that pectin-based polymers formed aggregates in aqueous solution with a unimodal distribution. Critical aggregation concentration (*cac*) for the hydrophobic pectin derivatives were determined using fluorescence spectroscopy. Atom force microscopy (AFM) images allowed for the investigation of the morphology of polymeric populations obtained in aqueous solution, consisting of flocs and aggregates for crude pectin and its hydrophilic derivatives and well-organized aggregates for lipophilic pectin derivatives. Antimicrobial activity, examined using the disc diffusion method, proved that all polymers were active against *Staphylococcus aureus* bacterium and *Candida albicans* yeast.

## 1. Introduction

Pectin is an anionic natural heteropolysaccharide, being the most abundant and multifunctional component of the cell wall of land plants [1,2,3]. It is biocompatible, biodegradable and non-toxic, having a high-molecular weight. Chemically, pectin contains α-(l–4)-d-galacturonic acid units, methyl–esterified at C-6 and O-acetylated at O-2 and/or O-3, interrupted by α-(1–2)-l-rhamnose units (“smooth regions”) and branched areas (“hairy regions”) consisting of rhamnogalacturonan I (RGI) and rhamnogalacturonan II (RGII) with lateral chains of galactan, arabinan, arabinogalactan and other complex segments. Pectin’s degree of methoxylation (DM) allowed for its classification in high methoxyl pectin (DM > 50) and low methoxyl pectin (DM < 50). Generally, pectin is extracted from citrus peel (orange, lemon, grapefruit and lime) and the pulp of different fruits. Other plant sources of pectin are sunflower head, potato, sugar beet, tomato and carrot. As a biomaterial, pectin is used in several applications such as tissue engineering [4], wound dressing [5], gene transfer [6], drug delivery [7], treatment of cancer [8], hyperglycaemia [9] and hypercholesterolemia [10]. Pectin is known as a thickener, stabilizer, coating and gelling agent in the food industry [11,12]. Pectin is also used to form edible films, foams and plasticizers [13,14]. A broad range of pectin derivatives could be prepared due to the occurrence of a high number of hydroxyl and carboxyl groups distributed along the polysaccharide main chain or on neutral sugars presented as side chains [15,16]. Pectin derivatives showed modified properties concerning solubility, lipophilicity and physicochemical and biological features compared to those of native polysaccharide. The derivatization of neat pectins was performed using substitution reactions like esterification [17], amidation [18], quaternization [19,20,21,22,23], thiolation [24], sulfation [25], oxidation [26], chain elongation (cross-linking [27], grafting [28]), etc.

Generally, the quaternization reaction of the polysaccharides has been carried out with commercially available reactants having preformed quaternary ammonium groups such as *N*-(3-chloro-2-hydroxypropyl)-*N*,*N*,*N*-trimethylammonium chloride (CHPTAC) or *N*-(glycidyl)-*N*,*N*,*N*-trimethylammonium chloride (GTAC) [19]. This procedure was used for the quaternization of many polysaccharides, such as starch [29], corn cob meal [30], cellulose [31], microcrystalline cellulose [32], nanofibrillated cellulose [33], hemicellulose [34], xylan [35], ganoderma lucidum glucan [36], dextran [37], tamarind kernel polysaccharide [38], chitosan [39,40], methylan [41], glucomannan [42], inulin [43], guar gum [44], gellan gum [45], konjak cashew gum [46], angico gum [47], etc. Unfortunately, the synthesis of preformed quaternization reagents is difficult and expensive and the reaction of the polysaccharides with this type of reagent needs an alkaline medium (NaOH), which can affect the supramolecular structure or even degrade the polysaccharides. Also, the polysaccharides can be quaternized by using a reaction mixture consisting of a tertiary amine and epichlorohydrin [48]. This one pot procedure is versatile because it allows the obtaining of numerous derivatives only by replacing the tertiary amine used in reaction. Dextran and carboxymethyl chitosan are among the polysaccharides used for the obtaining of quaternary ammonium derivatives by utilizing the above-mentioned protocol and the produced derivatives which have proven to have various applications. Thus, soluble quaternized polymers based on dextran demonstrate antimicrobial activity [48] while crosslinked dextrans exhibit adsorption ability of different sorbents such as bile salts [49,50] or dyes [51,52]. Quaternized carboxymethyl chitosan was utilized as one of the components of a hydrogel developed for the treatment of chronic wound infections [53].

The current study reports the synthesis of new citrus pectin-based polymers, bearing cationic pendant groups, created by the reaction between the polysaccharide and a quaternization mixture. The study also reports on the investigation of the structure and the arrangement of polymeric chains in aqueous solutions. The main goal of the work was the study of the antibacterial and antifungal activity of pectin and its quaternary ammonium derivatives, knowing from the literature that crude pectins have antimicrobial activity *per se.*

## 2. Materials and Methods

### 2.1. Materials

Pectin from citrus peel (PC)(galacturonic acid content ≥74%), epichlorohydrin (99%, GC), *N*,*N*-dimethyl-*N*-alkyl amine (purum, ≥98%) (alkyl = ethyl, butyl, benzyl, octyl, dodecyl), silver nitrate (ACS-reagent, >99%), sodium hydroxide (reagent grade, ≥98%) and HCl (ACS-reagent, 37%) were purchased from Sigma-Aldrich (Saint Louis, MO, USA). PC was purified via solubilization in ultrapure water, centrifugation, filtration, dialysis (12 KDa cut-off of dialysis bags) and recovered using lyophilization. Ultrapure water, with a conductivity of 0.055 μS/cm, was obtained with a Millipore purification system and used for the preparation of aqueous solutions. Microbial strains *S. aureus* ATCC 25923, *Escherichia coli* ATCC 25922, *Pseudomonas aeruginosa* ATCC 27853 and *C. albicans* ATCC 90028 were obtained from the Culture Collection of the Department of Microbiology, Faculty of Pharmacy, “Gr. T. Popa” University of Medicine and Pharmacy, Iasi, Romania.

### 2.2. Synthetic Procedure

PC, solved in ultrapure water (1 g polymer/20 mL pure water), reacted with a mixture of epichlorohydrin (ECH) and tertiary amine (*N*,*N*-dimethyl-*N*-alkyl amine) (alkyl = ethyl, butyl, benzyl, octyl, dodecyl), with the molar ratio between PC, ECH and tertiary amine being 1:8:9.

The preparation of the derivative of PC, obtained by amination with a mixture of *N*,*N*-dimethyl-*N*-ethyl amine and ECH, is given as an example: PC (2 g, 0.0105 saccharide units), dissolved in 40 mL ultrapure water, reacted with a mixture of *N*,*N*-dimethyl-*N*-ethyl amine (9.1 mL, 0.084 mol) and ECH (5.77 mL, 0.074 mol) under magnetic stirring at 70 °C for 6 h. The obtained product was precipitated in a methanol/acetone mixture (*v*/*v*)(1/1), then redissolved in water, filtrated, introduced into a dialysis bag (12 KDa cut-off) and dialyzed against 0.1 N HCl (24 h) and water until the conductivity of the dialyzate was close to that of ultrapure water. Finally, aqueous solutions of pectin derivative were recovered via freeze-drying. 

The degrees of substitution (DS) of pectin derivatives were found using elemental analysis (N%). The DS of the polymers, expressed in moles of side-chains per 100 α-(l-4)-galacturonic acid units (GA), was calculated with Equation (1):

(1) $$\mathrm{DS}=\frac{176\times N}{\left[\left(100\times 14\right)-\left(N\times MS\right)\right]}\times \;100,\;\mathrm{mol}/100\mathrm{GA},$$

where 176 is the molecular weight of GA unit; N is the nitrogen amount, in %, determined via elemental analysis; 14 is elemental mass for N; and *MS* is the molecular weight of the pendant ammonium group.

### 2.3. Methods

#### 2.3.1. Chemical Structure Characterization

^1^H- and ^13^C NMR spectra were obtained in D_2_O with a BrukerAvance NEO 400 MHz spectrometer (Rheinstetten, Germany) (400.1 MHz for ^1^H and 100.6 MHz for ^13^C nuclei) using sodium 3-(trimethylsilyl)-[2,2,3,3-d4]-1-propionate (TSP) as internal standard. FTIR spectra were recorded with a Bruker Vertex 70 (Ettlingen, Germany) spectrophotometer on KBr pellets. Elemental analysis (N%) was carried out by means of a CHNS 2400 II Perkin Elmer analyzer (Waltham, MA, USA).

#### 2.3.2. Conductometric Titration 

The carboxyl and methyl–ester groups contents of PC were found using the back titration method. For the determination of the content of carboxylic groups (mEq/g), 0.1 g PC was solved in ultrapure water, then 0.1 M NaOH solution was added in excess. The conductometric titration was achieved by using 0.1 N HCl solution. The graph representing the variation of the conductivity against HCl volume had two break points, the first corresponding to excess NaOH, the second coordinating to the neutralization of COONa groups. The HCl volume between the break points (*V*^1^*_HCl_*) was used for quantification of pectin carboxylic groups (*C_COOH_*) with Equation (2):

(2) $$C_{COOH}=\frac{V_{HCl}^{1}\times c_{HCl}^{1}}{m},$$

where *V^1^_HCl_* (L) is the volume of 0.1 N HCl solution used between the two break points; *c*^1^*_HCl_* (mol/L) is molar concentration of HCl solution; and *m* (g) is the amount of dry polysaccharide.

The sum of the content of carboxyl and methyl–ester groups (mEq/g) was calculated using the same reverse titration as described previously, with the mention that aqueous solutions of NaOH and HCl had 0.25 N. Under such conditions, ester groups are hydrolyzed to carboxyl groups. The content of pectin carboxylic and ester groups (*C_COOH+COOCH_*_3_) was found with Equation (3):

(3) $$C_{{COOH+COOCH}_{3}}=\frac{V_{HCl}^{2}\times c_{HCl}^{2}}{m},$$

where *V*^2^*_HCl_* (L) is volume of 0.25 N HCl solution used between the break points; *c*^2^*_HCl_* (mol/L) is molar concentration of HCl solution; and *m* (g) is the amount of dry PC.

#### 2.3.3. Capillary Viscometry Measurements

The viscosimetric behavior of PC and its derivatives was studied at 25 °C by using an Ubbehlode viscometer (0a type, capillary diameter of 0.53 mm) immersed in a thermostated bath at 25.0 ± 0.1 °C. Aqueous solutions of polymer concentrations (C_P_ = 0.025–0.5 g/dL), prepared in ultrapure water, were allowed to equilibrate for 24 h. The data were the average of duplicate measurements for each sample. [η] can be accurately estimated (error generally ~1%) using the Solomon-Ciuta equation (Equation (4)) [54], with specific (*η_sp_*) and relative viscosities (*η_rel_*) being determined by a single measurement at low concentration. The viscosity-average molar mass of PC was determined with the help of the Houwink–Kuhn–Sakurada equation (Equation (5)) [55]:

(4) $$[\eta ]=\frac{{\left[2\eta_{sp}-2ln\left(\eta_{rel}\right)\right]}^{1/2}}{c},$$

(5) $$[\eta ]=0.0174\times M_{w}^{0.84}\;(\mathrm{mL}/g),$$

#### 2.3.4. DLS Measurements

The average size, size distribution and ζ-potential of pectin and its derivatives were evaluated via DLS using a Zetasizer model Nano ZS, with red laser 633 nm He/Ne (Malvern Instruments, Malvern, UK) on 1 mg/mL polymer solution prepared in ultrapure water. The results were the average of triplicate measurements for each sample at room temperature (r.t.). The size of the polymers was determined using the methods of cumulants applied by intensity distribution.

#### 2.3.5. Fluorescence Measurements

The critical aggregation concentration (*cac*) of hydrophobic pectin derivatives was determined using pyrene as a fluorescence probe. Various *C*_P_, ranging from 4 mg/mL to 0.005 mg/mL, were prepared in vials by using pyrene aqueous solution (10^−6^ M). All of the mixtures were left at r.t. for 24 h to ensure that the cromophore was completely entrapped into polymeric hydrophobic microdomains. Pyrene was excited at 337 nm, and the excitation and emission slits were fixed at 5 and 3 nm, respectively. The emission spectra of pyrene were recorded in the range of 350–500 nm using a LS 55 PerkinElmer (Waltham, MA, USA) fluorescence spectrometer. The emission intensities, registered at 372 nm (I_1_) and 383 nm (I_3_), were used for the calculation of the polarity parameter (I_1_/I_3_).

#### 2.3.6. AFM

AFM experiments were carried out by depositing a drop of polymeric aqueous solution (1 mg/mL) onto a 1 × 1 cm freshly cleaved mica surface. Each sample was allowed to dry for 24 h at r.t. covered by a Petri dish to avoid dust. The surface images were obtained with an NTEGRA scanning probe microscope (NT-MDT Spectrum Instruments, Moscow, Russia) in AFM configuration. Rectangular silicon cantilevers NSG10 (NT-MDT, Moscow, Russia) with tips of a high aspect ratio (sharpened pyramidal tip with an angle of nearly 20°, a tip curvature radius of 10 nm and a height of 14–16 μm) were used in order to minimize convolution effects. All images were acquired in air, at r.t, in tapping mode, with a velocity of 6 mm/s. For image acquisition, the Nova v.19891 for Solver software was used. 

#### 2.3.7. Antimicrobial Activity

Antimicrobial activity of PC was examined using Gram-positive bacterium (*Staphylococcus aureus* ATCC 25923), Gram-negative bacteria (*E. coli* ATCC 25922, *P. aeruginosa* ATCC 27853) and a pathogenic yeast (*C. albicans* ATCC 90028). The antimicrobial susceptibility tests were performed using disc diffusion methods [56,57]. Mueller–Hinton agar (Oxoid) and Mueller–Hinton agar Fungi (Biolab) were inoculated with the suspensions of the tested microorganisms. Sterile stainless-steel cylinders (5 mm internal diameter; 10 mm height) were put on the agar surface in Petri plates. Then, 100 μL of the polymer, as 10 mg/mL aqueous solutions, were added into cylinders. The plates were kept 10 min at r.t. to obtain a homogeneous distribution of the compound in the medium and incubated at 35 °C for 24 h afterwards. As reference antimicrobial drugs were used commercially available discs containing Ciprofloxacin (5 μg/disc) and Nystatin (25 μg/disc). After incubation, the diameters of the inhibition zones were measured in mm, including disc size.

## 3. Results and Discussion

### 3.1. Synthesis of Quaternary Ammonium Derivatives of Pectin

**Scheme 1:**
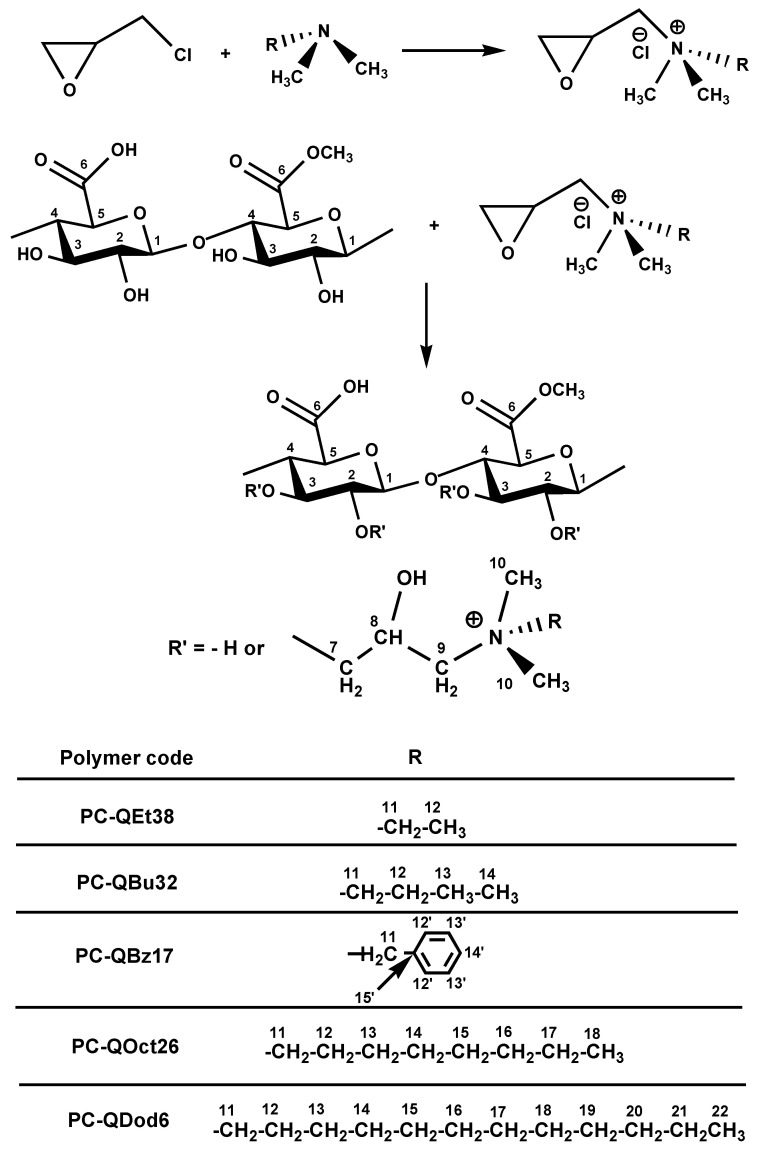
Synthetic route for PC-Q derivatives.

The derivatives of pectin (PC-Q) were acquired by the reaction between the polysaccharide and the quaternization mixture (tertiary amine + ECH), which allowed for the formation of well-controlled chemical structures, having quaternary ammonium pendant groups statistically distributed along the polysaccharide chain (Scheme 1). The tertiary amine (*N*,*N*-dimethyl-*N*-alkyl amine) (alkyl = ethyl, butyl, benzyl, octyl, dodecyl) and ECH reacted and formed, *in situ*, the quaternary ammonium reagent, which further attacked PC. The small excess of tertiary amine acted as a catalyst, improving the substitution degree. Thus, several quaternized pectins, with different characteristics and potential applications, can be obtained merely by changing the tertiary amine. The DS values of quaternary ammonium derivatives were determined by knowing % N (elemental analysis) and from ^1^H-NMR spectra. For the first technique, DS values were calculated with Equation (1). DS decreased with the augmentation of the length of alkyl substituent of the tertiary amine (*N*,*N*-dimethyl-*N*-alkyl amine) due to the decrease in water solubility of the tertiary amine used in synthesis and also due to the steric hindrance, in the case of bulky alkyl substituents like benzyl, octyl or dodecyl, exerted in amination reaction. The code for pectin derivatives was PC-QRX, where R is the substituent at amino group according to Scheme 1 [R = Et (ethyl), Bu (butyl), Bz (benzyl), Oct (octyl), Dod (dodecyl)] and X = DS, determined using elemental analysis (% N).

### 3.2. FTIR Analysis

**Figure 1:**
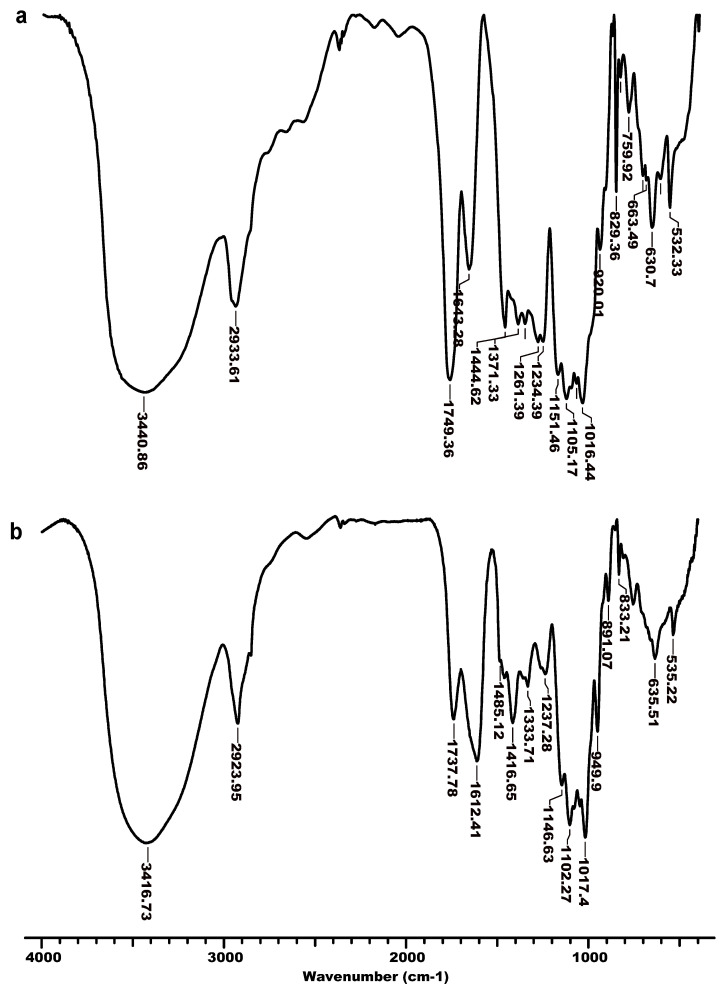
FTIR spectra of PC (**a**) and PC-QEt38 (**b**).

FTIR studies confirmed the formation of pectin derivatives having cationic side-chains. The FTIR spectra of the PC and one of its quaternized derivatives (PC-QEt38), chosen as an example, are depicted in Figure 1, while the FTIR spectral data for PC and all its derivatives are collected in Table 1. PC exhibited a wide band at 3440 cm^−1^, attributed to stretching vibration of O–H groups while the band at 2933 cm^−1^ was assigned to the stretching vibration of C–H groups [55,58]. The peak from 1749 cm^−1^ is characteristic to the C=O stretching vibrations of methyl–ester groups, whilst the peak from 1643 cm^−1^ was typical for stretching vibrations of COO^−^ groups [59]. FTIR spectra of quaternized derivatives of PC showed mutual peaks with PC at 3401–3419 cm^−1^, attributed to O–H stretching vibrations, and at 2923–2936 cm^−1^, characteristic to stretching vibrations of C–H groups [58]. FTIR spectra of pectin derivatives showed that the area of the peaks, corresponding to C=O stretching vibrations of methyl–ester groups (1738–1742 cm^−1^), decreased after amination while the area of the peaks, assigned to stretching vibrations of COO- groups (1608–1612 cm^−1^), increase. The cause was the partial hydrolysis of pectin ester groups during the synthesis, probably due to the excess of tertiary amine used in amination reaction. FTIR spectra of quaternized derivatives also showed the stretching vibrations corresponded to the methyl groups bound to quaternary nitrogen of pendant groups (1480–1485 cm^−1^) [58,59]. Furthermore, peaks of pectin derivatives, attributed to C–N stretching vibrations, could be seen at 1413–1416 cm^−1^ [60].

Another proof of pectin derivatives formation was the presence of –(CH_2_)_n_– (n > 5) rocking bands at 719–721 cm^−1^ in the FTIR spectra of PC-QOct27 and PC-QDod6 [61]. The “fingerprint’’ region of polysaccharides (between 800–1300 cm^−1^), containing skeletal C–O and C–C stretching vibrations bands of glycosidic bonds and pyranoid rings, respectively, could be seen in FTIR spectra of PC and its quaternary ammonium derivatives [62]. Due to the occurrence of benzyl substituent in PC-QBz17 structure, its FTIR spectrum revealed peaks were attributed to in-plane C–H bending (1040 cm^−1^), out-of-plane C–H bending (953 cm^−1^) and C–H oop (737 cm^−1^) (Table 1). The degree of methoxylation (DM) of PC and its quaternary ammonium derivatives (Table 2), calculated using their FTIR spectra, were expressed as the ratio between the peak area of methyl–esterified carboxyl groups and the sum of the peak areas of methyl–esterified carboxyl groups and free carboxyl groups COO^-^, according to Equation (6) [63]:

(6) $$\mathrm{DM}=\frac{\mathrm{peak}\;\mathrm{area}\;\mathrm{of}\;\mathrm{methyl}-\mathrm{esterified}\;\mathrm{carboxyl}\;\mathrm{groups}}{\;\mathrm{peak}\;\mathrm{area}\;\mathrm{of}\;\mathrm{methyl}-\mathrm{esterified}\;\mathrm{carboxyl}\;\mathrm{groups}\;+\mathrm{peak}\;\mathrm{area}\;\mathrm{of}\;\mathrm{free}\;\mathrm{carboxyl}\;\mathrm{groups}}\times \;100\%$$

The DM values for pectin derivatives were lower than those of PC due to a de-esterification reaction of the polysaccharide during its amination. MicrocalOriginPro^®^ 8.5 was the software used for the calculation of DM values.

### 3.3. ^1^H and ^13^C NMR Analysis

**Figure 2:**
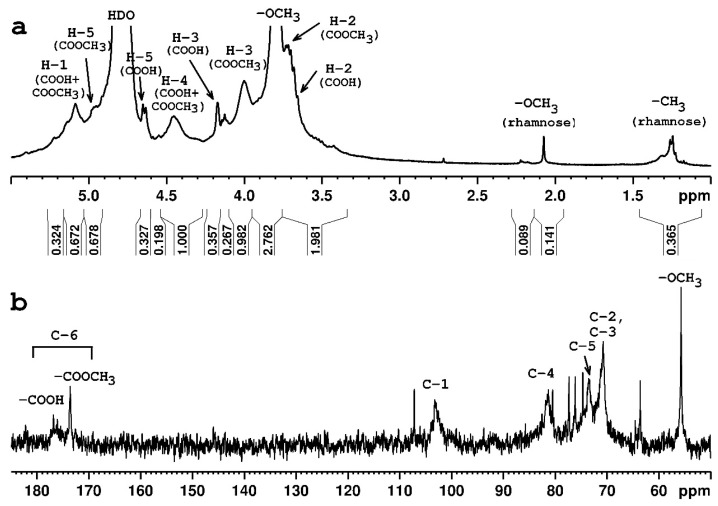
^1^H (**a**) and ^13^C (**b**) NMR (D_2_O) spectra of PC.

**Figure 3:**
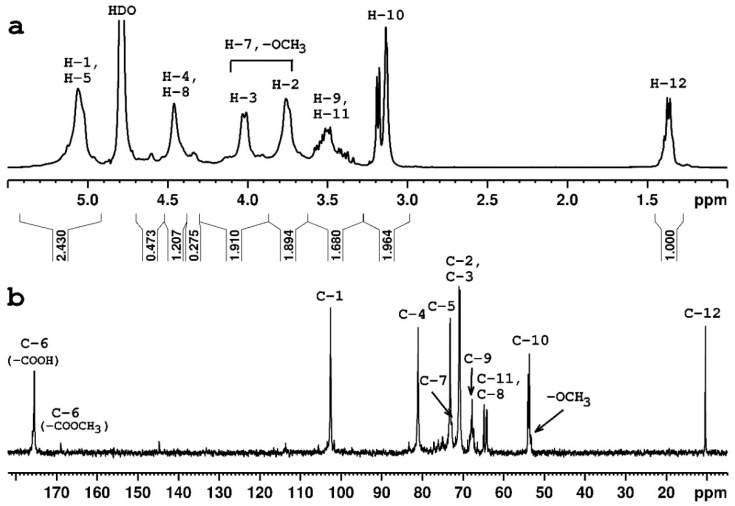
^1^H (**a**) and ^13^C (**b**) NMR (D_2_O) spectra of PC-QEt38.

1D NMR spectroscopy was used to prove the successful quaternization of PC. ^1^H and ^13^C NMR spectra corresponding to PC and one of its derivatives, PC-QEt38, given as an example, are shown in Figure 2 and Figure 3, respectively, whilst the 1D NMR spectra of the other quaternized derivatives of PC (Figures S1–S4) and the tables containing the chemical shifts of all derivatives (Tables S1–S5) are revealed in the Supplementary Material.

The DM % of PC, calculated with the help of FTIR spectroscopy (Section 3.1) and conductometric titration (Section 2.3.2), can be also determined using its ^1^H NMR spectrum (Figure 2a) (Table 2). DM was determined by taking into account the integral values of H-3 protons from un-esterified groups and H-4 from both un-esterified and methyl–esterified groups, according to Equation (7):

(7) $$\;\mathrm{DM}\;\left(\%\right)=\left[100-\left(\frac{I_{H3\left(\mathrm{COOH}\right)}}{I_{H4\left(\mathrm{COOH}+\mathrm{COOCH}3\right)}}\right)\times 100\right]\%$$

The carbon atoms and their corresponding hydrogen atoms of pectin and its quaternary ammonium derivatives from Figure 2, Figure 3 and Figures S1–S4 and Tables S1–S4 were numbered according to Scheme 1.

In ^1^H NMR spectrum of PC (Figure 2a) could be seen signals characteristic to methyl groups of l-rhamnose (1.21 ppm), O-2 linked rhamnose and O-2,4 linked rhamnose (1.32 ppm) [64]. In the range of 2.07–2.22 ppm, there were two signals derived from acetyl groups bound at O-2 and O-3 of GA units [65]. The presence of methyl–ester groups at C-6 from pectin structure determines different neighbors for H-2, H-3 and H-5 protons from the GA unit; therefore, signals in esterified and un-esterified units have different chemical shifts for these protons. Consequently, the signals for H-2, H-3 and H-5 protons from the ^1^H NMR spectrum of PC can have different chemical shifts. Thus, H-2 (3.71 ppm) and H-5 (4.96 ppm) protons in methyl–esterified GA units are downfield shifted compared with un-esterified ones (H-2 (3.65 ppm) and H-5 (4.64 ppm)), while H-3 (4.0 ppm) protons in esterified GA units are upfield shifted in comparison with un-esterified ones (H-3 (4.17 ppm)). The peaks at 5.08 and 4.45 ppm were attributed to H-1 and the respective H-4 from both methyl–esterified and un-esterified GA units, these two signals being overlapped. 

A large signal, corresponding to the CH_3_ of methyl–ester groups of GA, occurred at 3.80 ppm [64,66]. The values of the above-mentioned chemical shifts were close to those previously reported for other pectins extracted from citrus peels [67], pumpkin [64,68], the endocarp of Citrus depressa [64] and apple pomace [69].

In the ^13^C NMR spectrum of PC (Figure 2b), the peaks at 176.9 and 173.65 ppm were assigned to un-esterified and methyl–esterified C-6 carbons of GA, respectively, while the peaks at 103.21, 81.33 and 73.56 ppm were attributed to the C-1, C-4 and C-5 carbons of GA, respectively. The signals typical for C-2 and C-3 carbons are overlapped and could be seen at 70.7 ppm while the peak, belonging to CH_3_ carbon of methyl–ester group of GA, was found at 55.7 ppm. The values in ppm of the characteristic peaks of PC were in accordance with the values reported in the literature for other native pectins [70].

^1^H and ^13^C NMR spectra (Figure 3) of the derivative PC-QEt38, chosen as an example, proved the attachment of the quaternary ammonium groups to pectin backbone. The occurrence of cationic pendant group, carrying an ethyl substituent, was demonstrated by the presence of the signals assigned in the proton NMR spectrum (Figure 2a) to methyl protons H-10 (3.12–3.19 ppm) bound to quaternary nitrogen and methyl protons H-12 (1.35–1.39 ppm) from ethyl substituent. Methylene protons H-9 and H-11, bound to cationic nitrogen, are overlapped in the chemical shift region between 3.34 and 3.58 ppm. The most downfield shifted signal of R’ substituent (Scheme 1) could be observed for methine protons H-8 at 4.46 ppm, who were overlapped with the H-4 signal from the GA unit. The signals for methylene protons attached to oxygen atom from pectin (H-7) were found in the chemical shift regions between 3.75 and 4.03 ppm. The peaks assigned to protons H-1–H-5 from the GA backbone were found in the 3.7–5.2 ppm region, the values being in accordance with those given by the literature [64,67,68,69]. The ^1^H NMR spectra afforded the calculation of DS values for the derivatives of PC. For PC-QEt38, DS was determined using the integral value of the CH_3_ group belonging to the ethyl substituent bound to quaternary nitrogen (H-12) and that corresponding to the H-4 proton of the GA unit (Figure 3a). The integral value of H-8 protons was calculated by knowing the integral value of H-12 protons because the signal for H-4 protons was overlapped with that corresponding to H-8 protons. Thus, by considering the integral value of H-12 (three protons) to be equal to 1, the methine proton H-8 (one proton) from the same quaternary ammonium group has the integral value equal to 1/3 (0.333). DS, expressed in mol/100 GA units (mol %), was calculated with Equation (8):

(8) $$\;\mathrm{DS}\;=\frac{I_{H12}/3}{\left[(I_{H4}+I_{H8})-(I_{H12}/3)\right]}\times 100\left(\mathrm{mol}\;\%\right)$$

The obtained value (38 mol%) was similar to that determined from elemental analysis (Table 2). 

PC-QEt38 showed in its carbon NMR spectrum (Figure 3b) the peaks characteristic to un-esterified and esterified C-6 carbons from GA at 175.48 ppm and 168.97 ppm, respectively. Signals corresponding to carbons from quaternary ammonium pendant groups, namely C-12 (10.37 ppm), C-11 (64.82 ppm), C-9 (67.78 ppm), C-8 (64.22 ppm) and C-7 (72.82 ppm), could also be seen in Figure 3b. 

Mutual peaks with PC can be seen in PC-QEt38 carbon spectrum as follows: C-1 (102.58 ppm), C-2 (70.96 ppm), C-3 (70.73 ppm), C-4 (81.05 ppm) and C-5 (73.17 ppm). The values found for ^13^C-chemical shifts of PC and its derivatives are in agreement with those reported for other neat pectins [70] or quaternary ammonium derivatives of citrus pectin [19], dextran [48], konjac glucomannan [71], cellulose [72] or starch [73].

### 3.4. Viscosity Method

Viscometry allows for the determination of viscosity-average molar mass (M_v_) and [η] values of PC and its derivatives along with the evaluation of polymeric conformation and hydrodynamic volume in solution [48]. M_v_ was calculated with Houwink–Kuhn–Sakurada equation (Equation (5)) by knowing the [η] value, which was determined using the Solomon–Ciuta equation (Equation (4)), by a single measurement at low concentration (0.05 g/mL). [η] of PC (328.6 mL/g) falls within the range of values (325–600 mL/g) found for different industrially or in lab extracted citrus pectins [74], while the M_v_ value of PC (1.23 × 10^5^) was similar to the value reported in the literature for this commercial pectin [74]. The [η] values of quaternary ammonium derivatives decreased compared to those of PC, due to the partial depolymerization of polysaccharide chains during the syntheses of quaternary ammonium derivatives which occurred as a result of temperature and excess of tertiary amine (Table 3). Previous studies indicated a similar decrease in [η] for cationic derivatives, obtained by the quaternization with CHPTAC of different polysaccharides (konjac glucomannan [71], chitin [75] and hawthorn pectin [19]) due to the presence of NaOH, which degraded the polymeric structure. 

**Figure 4:**
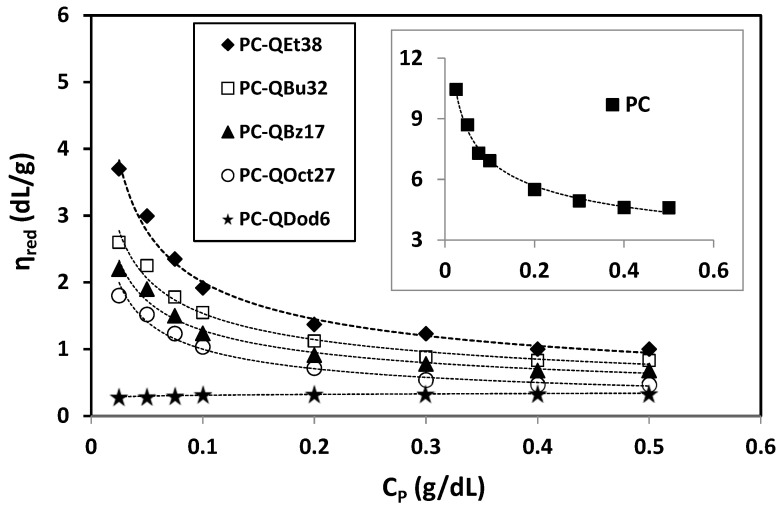
Huggins plots for the aqueous solutions of PC (inset) and its quaternary ammonium derivatives at r.t.

Capillary viscosity studies were carried out for pectin and its derivatives at low polymer concentrations (Cp ≤ 0.5 g/dL) in deionized water at r.t. in order to avoid the creation of viscous polymer solutions or even gels at higher polymeric concentrations [1]. Huggins plots for polymeric solutions are represented in Figure 4. PC and its hydrophilic derivatives containing ethyl, butyl and benzyl groups had higher viscosities compared to hydrophobic derivatives with octyl and dodecyl groups. PC and its derivatives (except the one having dodecyl substituents) showed a typical polyelectrolyte behavior (the reduced viscosity values increased with the decrease in C_P_). For PC-QDod6, the electrostatic repulsions were suppressed by the hydrophobic attractions between the very lipophilic groups. For PC, the increase in viscosity with the decrease in polymer concentration can be explained by the expansion of the polymer chains as a result of the electrostatic repulsions between the ionized carboxylate groups. The existing data indicated a dissociation in aqueous solution for HM pectins (65% DM) between 50–100% at a pH value in the range of 3.5–5.5, respectively [76]. This could explain the polyelectrolyte behavior of PC, which, having a similar DM value, has a pH = 3.7 at c_P_ = 0.5 g/dL. Polyelectrolyte behavior is preserved in case of quaternary ammonium derivatives, but it is less pronounced, as the new attached cationic groups neutralize neighbor carboxylic groups via internal carboxylate salts, which reduces the number of free carboxylic groups. Moreover, the polyelectrolyte chain expansion is reduced when more hydrophobic substituents are present, and for the derivative containing dodecyl groups, it is completely suppressed.

### 3.5. DLS Measurements

**Figure 5:**
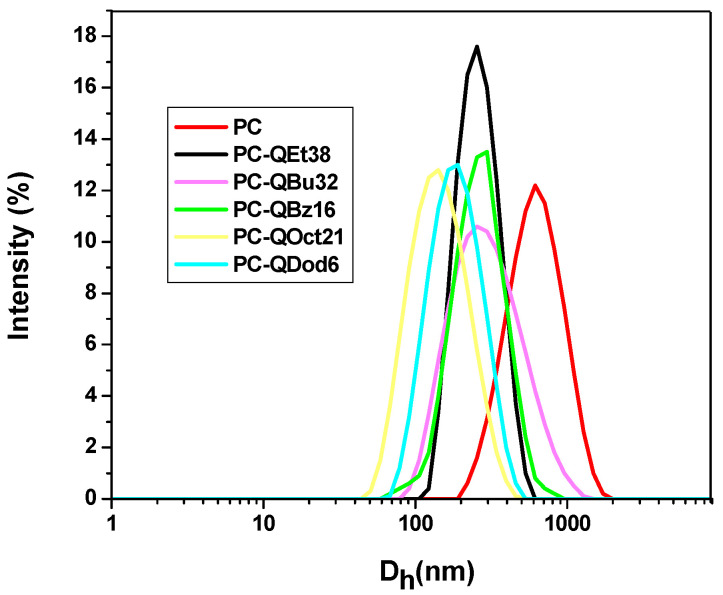
Hydrodynamic radius distribution of aggregates formed by pectin and its derivatives chains in aqueous solution (C_P_ = 0.1 g/dL).

DLS is a technique utilized for studying the diffusion behavior of macromolecules in solution. The hydrodynamic radii, D_h_, calculated from the diffusion coefficient, depends on the size and shape of macromolecules. The D_h_ of the polymeric chains in aqueous solution were determined for PC and its quaternary derivatives at a polymer concentration (C_P_) of 0.1 wt %. (Figure 5, Table 3).

Histograms obtained for pectin and PC-Q derivatives revealed a unimodal distribution. D_h_ of PC chains had about 561 nm (Figure 5, Table 3), which is in agreement with other D_h_ values obtained by DLS studies concerning native pectins [77]. The D_h_ values of the pectin derivatives were lower compared to that of PC due to the partial depolymerization of polysaccharide chains during the syntheses of its quaternized polymers [1]. The D_h_ values of quaternary ammonium derivatives depends on their DS and lipophilicity of pendant groups. The lower values were registered for PC-QOct and PC-QDod due to the hydrophobic interactions which occurred between polymeric side-chains having lipophilic substituents. Another parameter given by DLS analysis is zeta potential (ζ-potential), which is an indicator of the surface charge of particles. Its values for PC and pectin derivatives are shown in Table 3. The ζ-potential of PC had a negative value (−29.8 mV) due to pectins are anionic heteropolysaccharides composed of GA units in their main chain. The zeta potential values of PC-Q derivatives still had negative values but were lower in absolute value compared to those of PC because of the occurrence of permanent quaternary ammonium pendant groups, which make pectin derivatives act like polyampholytes. The internal salts formed between the anionic carboxylic groups and the attached quaternary ammonium unities behave like neutral entities, leading to a zeta potential close to the neutralization value.

### 3.6. Fluorescence Studies

**Figure 6:**
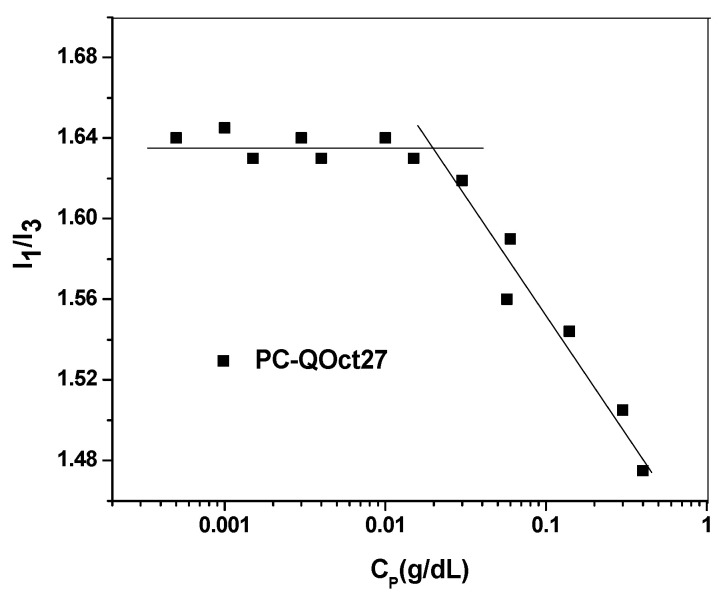
Variation of the polarity parameter with the polymer concentration for PC-QOct27.

Pyrene (Py) was used as free chromophore in fluorescence spectroscopy studies for the examination of the self-aggregation ability of hydrophobic quaternary ammonium derivatives of pectin (PC-QOct27 and PC-QDod6) in aqueous media. The polarity parameter (I_1_/I_3_), the variation in the ratio of intensity of the first (372 nm) (I_1_) to the third (383 nm) (I_3_) vibronic peaks of Py, is relatively sensitive to the polarity of microenvironment where the pyrene is placed. Figure 6 revealed the variation of Py polarity parameter with C_P_ for PC-QOct27. 

At low C_P_, the values of I_1_/I_3_ were almost unchanged, but with the augmentation of C_P_, the polarity parameter values decreased because of the self-assembly ability of lipophilic derivatives, which formed hydrophobic microdomains. Py molecules, retained by hydrophobic interactions in polymeric microdomains, sensed the polarity of the microenvironment, the cromophore polarity parameter being lower in less polar media [78]. The *cac* for PC-QOct27 and PC-QDod6 were determined to be 0.0198 g/dL (Figure 6) and 0.0226 g/dL, respectively, by the interception of two straight lines. These values indicate a moderate tendency to self-aggregation, which is expected for ionic amphiphilic polymers. It can be observed that *cac* values (g/dL) decreased with the augmentation of DS with lipophilic pendant groups.

### 3.7. AFM Technique

The atomic force microscope is a scanning probe microscope, which allows magnification analogous to that of electronic microscopy but obtainable under the more natural conditions used for light microscopy. The high resolution of AFM allowed for the study of dimensions and morphological arrangement of PC and its macromolecular derivatives. 

**Figure 7:**
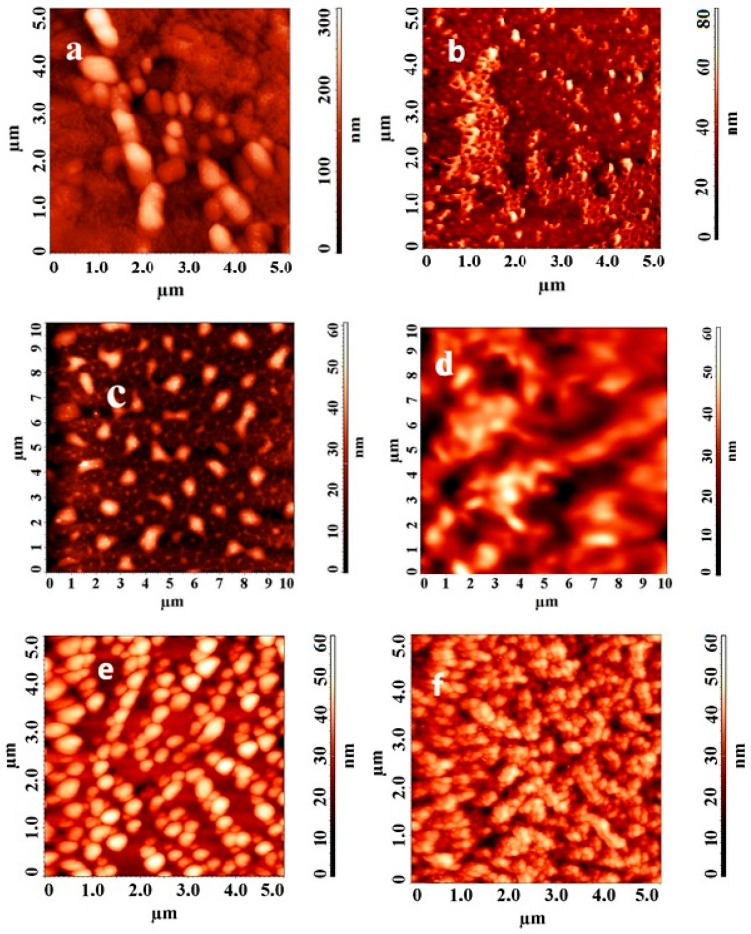
AFM topographical 2D images of PC (**a**), PC-QEt38 (**b**), PC-QBut32 (**c**), PC-QBz17 (**d**), PC-QOct27 (**e**), PC-QDod6 (**f**) drop-deposited onto mica and visualized in tapping mode.

AFM topographical 2D images for PC showed a mixed population of flocs and ovoidal aggregates (Figure 7a).

The maximum height of the aggregates was 300 nm, while their width was in the range of 280–480 nm. Similar dimensions could be seen for other neat pectins, with their aggregates occurring even at high dilution, which suggests that they are not simply superposition or entanglements of polymeric chains but are complexes formed due to intra- and intermolecular segment–segment interactions (hydrogen bonds and hydrophobic interactions) [79,80]. Width dimensions for the samples should be analyzed with extreme caution because of the “probe broadening” of AFM technique that overestimated the diameter of the molecules [79]. Pectin molecules were oriented on different directions due to their alignment along the crystal lattice of the mica surface [81]. Macromolecules have a polymeric backbone with no apparent branches. A possible explanation is that RGI, which contains the side chains, might not adsorb onto mica as well as the homogalacturonan regions because of a lower electric charge [81]. AFM topographical 2D images of pectin derivatives (Figure 7b–f) revealed the occurrence of similar structures, having smaller sizes of their height and width compared to that of pectin, due to both partial depolymerization and the presence of hydrophobic associations. 

A combined population of flocs and aggregates could be seen for hydrophilic derivatives of PC, the polymer containing benzylic groups having the bulkiest structures due to the presence of voluminous aromatic pendant groups, which can interact by intra- and intermolecular hydrophobic and π–π bonds. Better defined aggregates and higher aggregates density can be observed for the compounds having octyl and dodecyl pendant groups due to the occurrence of hydrophobic association between lipophilic side chain, which favor intra- and intermolecular self-associations.

### 3.8. Antimicrobial Activity

The main aim of the work was the study of antimicrobial activity of PC and its derivatives because the literature revealed that native pectins have antimicrobial activity *per se.* All the compounds were evaluated against a Gram-positive bacterium (*S. aureus* ATCC 25923), two Gram-negative bacteria (*E. coli* ATCC 25922, *P. aeruginosa* ATCC 27853) and a pathogenic yeast (*C. albicans* ATCC 90028). Antimicrobial susceptibility tests were used for the determination of polymeric antimicrobial activity, the inhibition zone diameters being showed in Table 4. Ciprofloxacin and Nystatin were used as positive controls for testing polymeric antimicrobial and antifungal activity, respectively. All tested products proved antimicrobial activity against Gram-positive bacterium and pathogenic yeast but did not demonstrate Gram-negative bacteria growth inhibition for the used concentration (10 mg/mL). The results can be explained by taking into account that Gram-positive bacteria are more accessible to polymeric compounds than Gram-negative bacteria due to their peptidoglycan layer, located outside the plasma membrane, which can be simply crossed by different biocides while for Gram-negative bacteria, the passive diffusion of products is extremely difficult due to the occurrence of the peptidoglycan layer between the inner and the outer cell membranes [82,83].

PC showed antimicrobial activity against *S. aureus* and *C. albicans* (Table 4), confirming the results obtained by previous studies which indicated that crude pectins are broad-spectrum antimicrobials able to kill bacteria, yeasts and non-filamentous fungi, although their mechanisms of action are still not understood [84,85].

Ionic and hydrophobic interactions between the derivatives of pectin and the outer phospholipid membrane of the microorganism generated several flaws in the membrane unity, with the waste of cytoplasmic elements determining the death of the pathogens. The forces of ionic attraction took place between the positive permanent charge of polymeric quaternary ammonium pendant groups and negatively-charged outer membrane of pathogen, whilst the lipophilic forces occurred between their hydrophobic segments. The best antimicrobial results were obtained for the derivatives having alkyl pendant groups with a medium length (benzyl and octyl) which are able to interact with the pathogens because of the occurrence of both ionic and hydrophobic interactions between the polymers and the lipid bilayer of microorganisms’ cell membrane. Polymers carrying short (ethyl, butyl) and long (dodecyl) alkyl substituents at cationic pendant groups showed reduced antimicrobial activity, probably due to the absence of hydrophobic interactions in the first case, and the difficult penetration of the biocide into the cell membrane of bacteria or yeast in the second case, as the hydrophobes are hidden inside dense microdomains. All polymers proved to be less active than the compounds used as positive controls (Table 4). Quaternary ammonium derivatives of other pectins also revealed antipathogenic activity [19].

## 4. Conclusions

Quaternary ammonium derivatives of PC were successfully synthesized by the reaction of the polysaccharide with a mixture of tertiary amine and ECH in aqueous solution. FTIR and 1D (^1^H and ^13^C) NMR studies confirmed the formation of pectin derivatives. DS values were found by elemental analysis while the contents of carboxylic and ester groups were determined via conductometric titration. The viscosity method was used for the determination of viscosity–average molar mass of PC, the [η] values of all compounds and for the study of viscometric behavior. So, a typical polyelectrolyte behavior could be seen for all polymers with the exception of hydrophobic pectin derivative carrying dodecyl pendant groups. DLS studies revealed that pectin-based polymers were able to form aggregates in aqueous medium with a unimodal distribution. AFM images revealed the occurrence of a mixed population of flocs and aggregates for PC and its hydrophilic derivatives, and well-defined aggregates for hydrophobic pectin derivatives. PC and its derivatives showed antimicrobial activity against *S. aureus* bacterium and *C. albicans* yeast.

## Figures and Tables

**Table 1:** FTIR spectral data of PC and its derivatives.

Type of Bond	Polymer Code					
	PC	PC-QEt38	PC-QBut32	PC-QBz17	PC-QOct27	PC-QDod6
O–H	3440	3416	3416	3409	3401	3417
C–H_(aliphatic)_	2933	2923	2923	2936	2927	2923
C=O_ester_	1749	1738	1741	1742	1741	1742
C=O_acid (ionic form)_	1643	1612	1612	1608	1611	1609
CH_3_–N^+^	-	1485	1485	1483	1480	1485
CH_3_–(CH_2_)_n_–CH_2_– N^+^	-	-	-	-	721	719
C–N	-	1416	1416	1413	1414	1413
C–O (glycosidic)	1105	1084	1084	1100	1099	1102
C–C (pyranoid ring)	1016	1017	1017	1021	1020	1021
C–H (aromatic)	-	-	-	1040/953/737	-	-

**Table 2:** DM(%) of PC and DS (mol %) of PC-Q derivatives determined by different analyses.

Polymer Code		PC	PC-QEt38	PC-QBu32	PC-QBz17	PC-QOct27	PC-QDod6
DM (%)	FTIR	62.7	46.4	48.1	52.2	50.1	53.4
	^1^H NMR	64.3	-	-	-	-	-
	conductometric titration	64.9	-	-	-	-	-
DS (mol %)	% N	-	38	32	17	27	6
	^1^H NMR	-	38	32	17	27	5.6

**Table 3:** D_h_, ζ-potential and [η] values of PC and its derivatives.

Polymer Code	PC	PC-QEt38	PC-QBu32	PC-QBz17	PC-QOct27	PC-QDod6
D_h_ (nm)	561	263	302	352	221	150
ζ-potential (mV)	−29.8	−4.4	−1.0	−0.8	−2.6	−10.1
[η] (mL/g)	328.6	111.3	93.9	80.6	63.2	39.3

**Table 4:** Antimicrobial activity of PC and its quaternary ammonium derivatives.

Polymeric Code	*S. aureus * ATCC 25923	*E. coli* ATCC 25922	*P. aeruginosa* ATCC 27853	*C. albicans* ATCC 90028
PC	11	-	-	11
PC-QEt38	12	-	-	14
PC-QBu32	14	-	-	12
PC-QBz17	17	-	-	15
PC-QOct27	19	-	-	16
PC-QDod6	12	-	-	11
Ciprofloxacin	34	35	33	-
Nystatin	-	-	-	25

## Data Availability

Data are contained within the article and supplementary materials.

## Supplementary Materials

The following supporting information can be downloaded at: https://www.mdpi.com/article/10.3390/polym15234492/s1, Figure S1: ^1^H (up) and ^13^C (down) NMR spectra of PC-QBut32 in D_2_O, with signals assignment; Figure S2: ^1^H (up) and ^13^C (down) NMR spectra of PC-QBz17 in D_2_O, with signals assignment; Figure S3: ^1^H (up) and ^13^C (down) NMR spectra of PC-QOct27 in D_2_O, with signals assignment; Figure S4: ^1^H (up) and ^13^C (down) NMR spectra of PC-QDod6 in D_2_O, with signals assignment; Table S1: ^1^H and ^13^CNMR chemical shift values for PC and PC-QEt38; Table S2: ^1^H and ^13^C NMR chemical shift values for PC-QBut32; Table S3: ^1^H and ^13^C NMR chemical shift values for PC-QBz17; Table S4: ^1^H and ^13^C NMR chemical shift values for PC-QOct27; Table S5: ^1^H and ^13^C NMR chemical shift values for PC-QDod6.
