# Supplementary material for: New Quaternary Ammonium Derivatives Based on Citrus Pectin
Source: Polymers (Basel). 2023 Nov 22;15(23):4492. doi: 10.3390/polym15234492 (PMC10707904; doi:10.3390/polym15234492)
Supplement: Supplementary file 1 [file polymers-15-04492-s001.zip › polymers-2692844-supplementary.pdf]

Supplementary Material

New quaternary ammonium derivatives based on citrus pectin

Magdalena-Cristina Stanciu[1,]*, Marieta Nichifor[1], Gabriela-Liliana Ailiesei[1], Irina Popescu[1], Gabriela-Elena Hitruc[1], Luminita Ghimici[1], Cristina G. Tuchilus[2]

[1]"Petru Poni" Institute of Macromolecular Chemistry, 41A, Gr. Ghica-Voda Alley, Iasi, 700487, Romania; cstanciu@icmpp.ro (M.-C.S.); nichifor@icmpp.ro (M.N.); gdarvaru@icmpp.ro (G.-L.A.); ipopescu@icmpp.ro (I.P.); gabihit@icmpp.ro (G.-E.H.); lghimici@icmpp.ro (L.G.)

[2]"Grigore T. Popa" University of Medicine and Pharmacy, Faculty of Medicine, 16, University Street, Iasi, 700115 Romania; ctuchilus@yahoo.com (C.G.T.)

* Correspondence: cstanciu@icmpp.ro

Table S1. ^{1}H and ^{13}CNMR chemical shift values for **PC** and **PC-QEt38**

Type of atom	Polymer code	Type of atom	Polymer code
	**PC**		**PC-QEt38**
**H-1** (COOH+$COOCH_3$)	5.08	**H-1**	5.06
**H-2** (COOH)	3.65	**H-2**	3.76
**H-2** ($COOCH_3$)	3.71	**H-3**	4.00
**H-3** (COOH)	4.17	**H-4**	4.46
**H-3** ($COOCH_3$)	4.00	**H-5**	5.06
**H-4** (COOH+$COOCH_3$)	4.45	**H-6**	-
**H-5** (COOH)	4.64	**H-7**	3.75-4.03
**H-5** ($COOCH_3$)	4.96	**H-8**	4.46
**H-6**	-	**H-9**	3.34-3.58
**-OCH_3**	3.80	**H-10**	3.12-3.19
		**H-11**	3.34-3.58
		**H-12**	1.35-1.39
		**-OCH_3**	3.76
**C-1**	103.21	**C-1**	102.58
**C-2**	70.75	**C-2**	70.96
**C-3**	70.75	**C-3**	70.73
**C-4**	81.33	**C-4**	81.05
**C-5**	73.56	**C-5**	73.17
**C-6** (COOH)	176.90	**C-6**(COOH)	175.48
**C-6** ($COOCH_3$)	173.65	**C-6**($COOCH_3$)	168.97
**-OCH_3**	55.70	**C-7**	72.82
		**C-8**	64.22
		**C-9**	67.78
		**C-10**	54.10
		**C-11**	64.82
		**C-12**	10.37
		**-OCH_3**	53.36

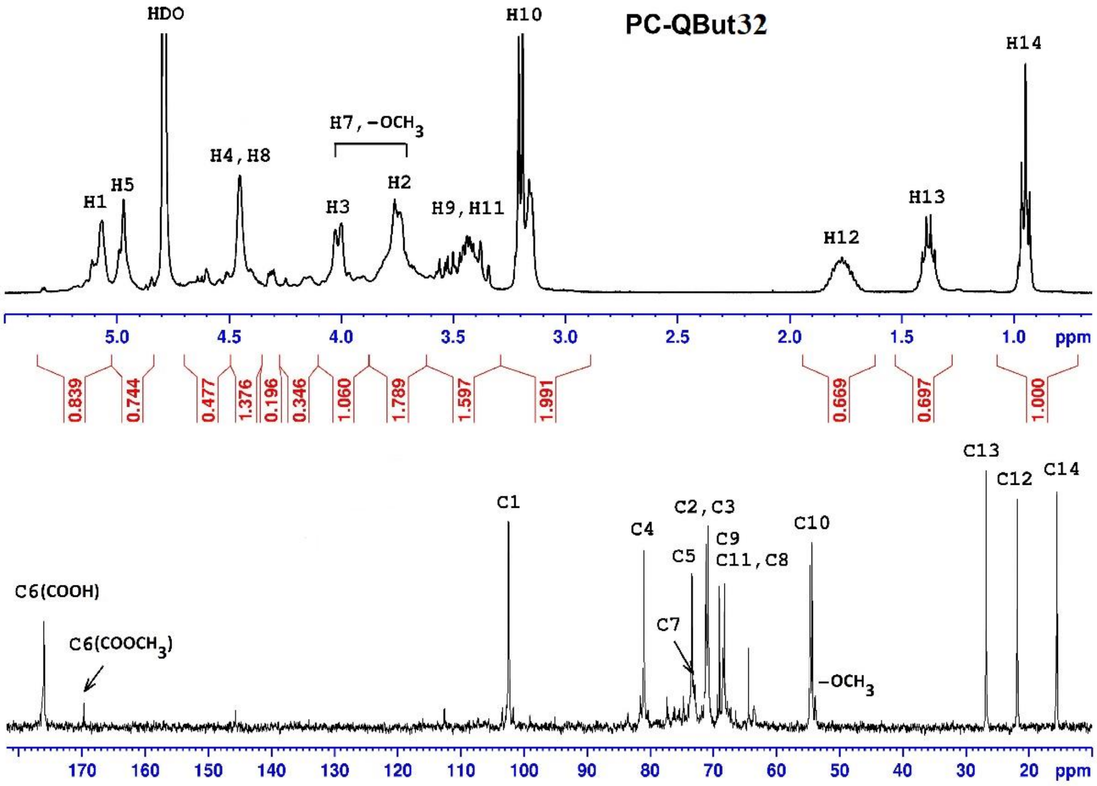

Figure S1. ^{1}H (up) and ^{13}C (down) NMR spectra of PC-QBut32 in D_2O, with signals assignment.

Table S2. ^{1}H and ^{13}C NMR chemical shift values for PC-QBut32

Polymer code	Chemical shift values, δ (ppm)			
	^{1}H NMR		^{13}C NMR	
	Hydrogen atom	δ (ppm)	Carbon atom	δ (ppm)
	**H-1**	5.06	**C-1**	102.46
	**H-2**	3.73	**C-2**	71.15
	**H-3**	3.99	**C-3**	70.84
	**H-4**	4.45	**C-4**	81.02
	**H-5**	4.97	**C-5**	73.40
	**H-6**	-	**C-6**(COOH)	176.02
	**H-7**	3.73-4.02	**C-6**($COOCH_3$)	169.72
**PC-QBut32**	**H-8**	4.40	**C-7**	72.86
	**H-9**	3.34-3.57	**C-8**	68.25
	**H-10**	3.15-3.20	**C-9**	69.03
	**H-11**	3.34-3.57	**C-10**	54.35
	**H-12**	1.76	**C-11**	68.48
	**H-13**	1.35-1.40	**C-12**	21.83
	**H-14**	0.92-0.96	**C-13**	26.79
	**-OCH_3**	3.76	**C-14**	15.59
			**-OCH_3**	53.92

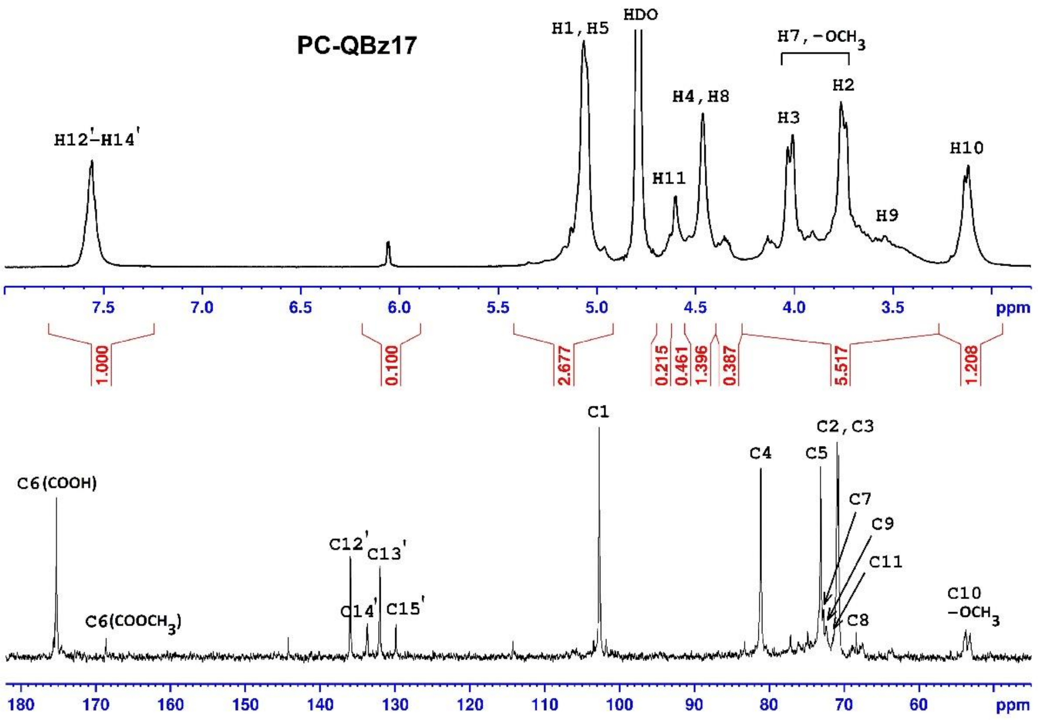

Figure S2. ^{1}H (up) and ^{13}C (down) NMR spectra of PC-QBz17 in D_2O, with signals assignment.

Table S3. ^{1}H and ^{13}C NMR chemical shift values for PC-QBz17

**Polymer code**	**Chemical shift values, δ (ppm)**			
	**^{1}H NMR**		**^{13}C NMR**	
	**Hydrogen atom**	**δ (ppm)**	**Carbon atom**	**δ (ppm)**
	**H-1**	5.06	**C-1**	102.71
	**H-2**	3.73	**C-2**	70.91
	**H-3**	4.00	**C-3**	70.73
	**H-4**	4.46	**C-4**	81.12
	**H-5**	5.06	**C-5**	73.11
	**H-6**	-	**C-6**(COOH)	175.24
	**H-7**	3.73-4.03	**C-6**($COOCH_3$)	168.62
**PC-QBz17**	**H-8**	4.53	**C-7**	72.75
	**H-9**	3.54	**C-8**	68.35
	**H-10**	3.11-3.13	**C-9**	72.40
	**H-11**	4.60	**C-10**	53.71
	**H-12’**	7.56	**C-11**	71.49
	**H-13’**	7.56	**C-12’**	135.96
	**H-14’**	7.56	**C-13’**	131.99
	**-OCH_3**	3.76	**C-14’**	133.67
			**C-15’**	129.89
			**-OCH_3**	53.12

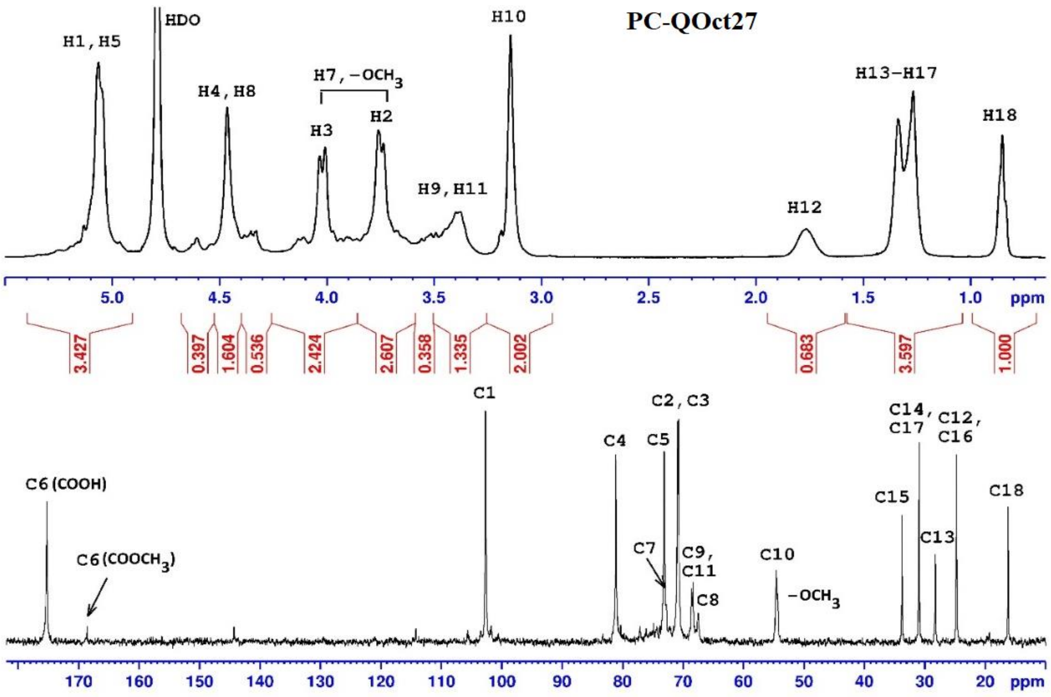

Figure S3. ^{1}H (up) and ^{13}C (down) NMR spectra of PC-QOct27 in D_2O, with signals assignment.

Table S4. ^{1}H and ^{13}C NMR chemical shift values for PC-QOct27

**Polymer code**	**Chemical shift values, δ (ppm)**			
	**^{1}H NMR**		**^{13}C NMR**	
	**Hydrogen atom**	**δ (ppm)**	**Carbon atom**	**δ (ppm)**
**PC-QOct26**	**H-1**	5.06	**C-1**	102.68
	**H-2**	3.73	**C-2**	70.90
	**H-3**	4.00	**C-3**	70.72
	**H-4**	4.46	**C-4**	81.11
	**H-5**	5.06	**C-5**	73.11
	**H-6**	-	**C-6**(COOH)	175.25
	**H-7**	3.75-4.03	**C-6**($COOCH_3$)	168.62
	**H-8**	4.46	**C-7**	72.74
	**H-9**	3.37-3.56	**C-8**	67.48
	**H-10**	3.14-3.18	**C-9**	68.61
	**H-11**	3.37-3.56	**C-10**	54.59
	**H-12**	1.76	**C-11**	68.35
	**H-13 – H-17**	1.26-1.33	**C-12**	24.77
	**H-18**	0.85	**C-13**	28.23
	**-OCH_3**	3.76	**C-14**	30.92
			**C-15**	33.75
			**C-16**	24.77
			**C-17**	30.92
			**C-18**	16.19
			**-OCH_3**	54.33

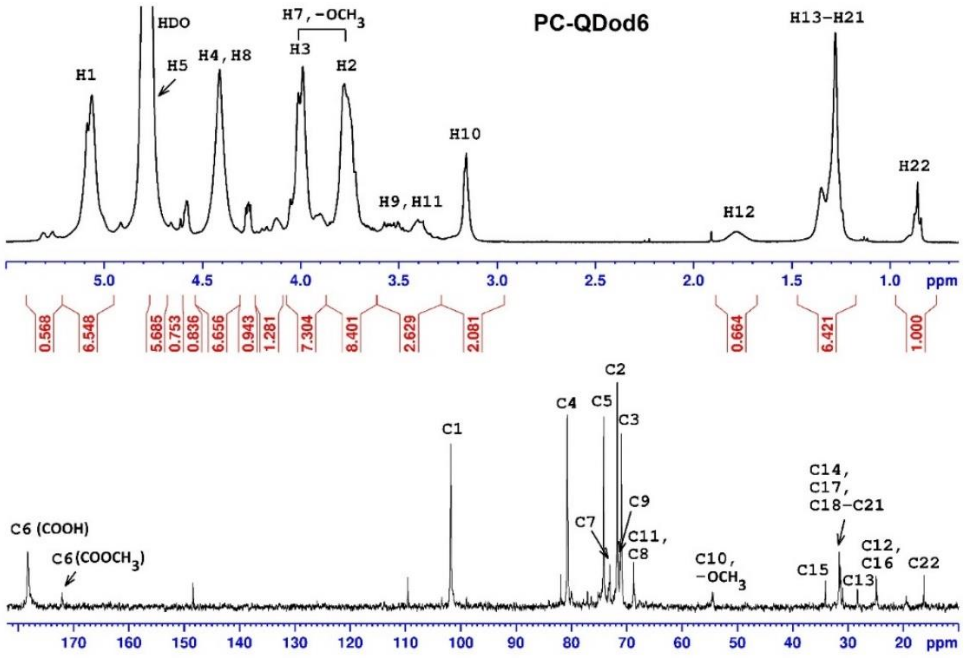

Figure S4. ^{1}H (up) and ^{13}C (down) NMR spectra of PC-QDod6 in D_2O, with signals assignment.

Table S5. ^{1}H and ^{13}C NMR chemical shift values for PC-QDod6

Polymer code	Chemical shift values, δ (ppm)			
	^{1}H NMR		^{13}C NMR	
	Hydrogen atom	δ (ppm)	Carbon atom	δ (ppm)
PC-QDod6	**H-1**	5.06	**C-1**	101.83
	**H-2**	3.76	**C-2**	71.71
	**H-3**	3.98	**C-3**	70.99
	**H-4**	4.41	**C-4**	80.76
	**H-5**	4.76	**C-5**	74.17
	**H-6**	-	**C-6**(COOH)	178.27
	**H-7**	3.76-4.01	**C-6**($COOCH_3$)	172.03
	**H-8**	4.41	**C-7**	73.06
	**H-9**	3.37-3.57	**C-8**	68.77
	**H-10**	3.15	**C-9**	71.24
	**H-11**	3.37-3.57	**C-10**	54.59
	**H-12**	1.77	**C-11**	68.77
	**H-13 – H-21**	1.27-1.35	**C-12**	24.91
	**H-22**	0.85	**C-13**	28.28
	**-OCH_3**	3.77	**C-14, C-17, C-18 – C21**	30.97-31.60
			**C-15**	34.06
			**C-16**	24.78
			**C-22**	16.29
			**-OCH_3**	54.34
